# A machine learning algorithm to differentiate bipolar disorder from major depressive disorder using an online mental health questionnaire and blood biomarker data

**DOI:** 10.1038/s41398-020-01181-x

**Published:** 2021-01-12

**Authors:** Jakub Tomasik, Sung Yeon Sarah Han, Giles Barton-Owen, Dan-Mircea Mirea, Nayra A. Martin-Key, Nitin Rustogi, Santiago G. Lago, Tony Olmert, Jason D. Cooper, Sureyya Ozcan, Pawel Eljasz, Grégoire Thomas, Robin Tuytten, Tim Metcalfe, Thea S. Schei, Lynn P. Farrag, Lauren V. Friend, Emily Bell, Dan Cowell, Sabine Bahn

**Affiliations:** 1 Department of Chemical Engineering and Biotechnology, University of Cambridge, Cambridge, UK; 2 Psyomics Ltd, Cambridge, UK; 3 SQU4RE, Roeselare, Belgium; 4 Metabolomic Diagnostics, Little Island, Cork Ireland; 5 Present Address: Princeton Neuroscience Institute, Princeton University, Princeton, New Jersey USA; 6 Present Address: University of California San Diego School of Medicine, San Diego, California USA; 7 Present Address: Owlstone Medical Ltd, Cambridge, UK; 8 Present Address: Department of Chemistry, Middle East Technical University, Ankara, Turkey; 9 Present Address: KPMG UK, London, UK

**Keywords:** Bipolar disorder, Depression, Diagnostic markers

## Abstract

The vast personal and economic burden of mood disorders is largely caused by their under- and misdiagnosis, which is associated with ineffective treatment and worsening of outcomes. Here, we aimed to develop a diagnostic algorithm, based on an online questionnaire and blood biomarker data, to reduce the misdiagnosis of bipolar disorder (BD) as major depressive disorder (MDD). Individuals with depressive symptoms (Patient Health Questionnaire-9 score ≥5) aged 18–45 years were recruited online. After completing a purpose-built online mental health questionnaire, eligible participants provided dried blood spot samples for biomarker analysis and underwent the World Health Organization World Mental Health Composite International Diagnostic Interview via telephone, to establish their mental health diagnosis. Extreme Gradient Boosting and nested cross-validation were used to train and validate diagnostic models differentiating BD from MDD in participants who self-reported a current MDD diagnosis. Mean test area under the receiver operating characteristic curve (AUROC) for separating participants with BD diagnosed as MDD (*N* = 126) from those with correct MDD diagnosis (*N* = 187) was 0.92 (95% CI: 0.86–0.97). Core predictors included elevated mood, grandiosity, talkativeness, recklessness and risky behaviour. Additional validation in participants with no previous mood disorder diagnosis showed AUROCs of 0.89 (0.86–0.91) and 0.90 (0.87–0.91) for separating newly diagnosed BD (*N* = 98) from MDD (*N* = 112) and subclinical low mood (*N* = 120), respectively. Validation in participants with a previous diagnosis of BD (*N* = 45) demonstrated sensitivity of 0.86 (0.57–0.96). The diagnostic algorithm accurately identified patients with BD in various clinical scenarios, and could help expedite accurate clinical diagnosis and treatment of BD.

## Introduction

Mood disorders are devastating psychiatric conditions which impose substantial burdens to individuals, healthcare systems and economies. Major depressive disorder (MDD) and bipolar disorder (BD) are two of the most common mood disorders and affect ~16.6% and 3.9% of the global population, respectively, throughout their lifetime^1^. In 2017 alone, about 163 million people (2.1% of global population) suffered from MDD and 46 million (0.6%) were affected by BD, accounting for 32.8 million years lived with disability (YLDs) in the case of MDD and 9.3 million YLDs for BD^2^. These numbers have been steadily increasing since the 1990s^2^ and both conditions are currently among the 20 leading causes of disability worldwide, with MDD ranked 2nd and BD 17th^3^. In England, the direct economic burden of managing mood disorders, encompassing healthcare, informal care and justice system services, is estimated at £1.68 billion annually for depression and £1.64 billion for bipolar spectrum disorders, while indirect costs associated with lost work productivity amount to £5.82 billion and £3.57 billion, respectively, and are expected to grow^4^.

A large proportion of this burden is caused by incorrect or late diagnosis and treatment of BD and MDD^5^, and could be significantly reduced by means of early interventions^4^. Although BD can be distinguished from MDD by the intermittent occurrence of manic (BD I) or hypomanic (BD II) episodes, these often remain undiagnosed as patients are more likely to seek medical help during a depressive episode^6^. In turn, because depressive episodes in BD are indistinguishable from those in MDD, BD is often misdiagnosed as MDD, even if the depressive symptoms were preceded by a manic/hypomanic episode. In fact, ~37% of patients with BD who present after their first manic/hypomanic episode are nonetheless misdiagnosed as having MDD^7^. Overall, it is estimated that at least 19% of individuals experiencing a major depressive episode have BD^8^, and that ~40% of patients with BD are initially diagnosed with MDD^7,9^, with the average delay in BD diagnosis ranging from 5.7 to 7.5 years^10,11^. As a result, misdiagnosed patients with BD are often incorrectly treated with antidepressants, which can aggravate the disease and worsen the outcomes^12^.

The correct diagnosis of BD and MDD is further impeded by the unknown aetiology of these conditions and the lack of objective diagnostic measures. Diagnosing BD and MDD relies primarily on assessing patient self-reported symptoms in accordance to state-of-the-art diagnostic manuals, such as the Diagnostic and Statistical Manual of Mental Disorders, 5th Edition (DSM-5)^13^ or the International Statistical Classification of Diseases and Related Health Problems, 11th Revision (ICD-11)^14^. While structured psychiatric interviews are considered a gold standard for mental health disorder diagnosis, their systematic use in primary care, where the majority of MDD diagnoses are made, can be hindered by factors such as availability of qualified staff, inter-rater variability and time constraints^15^. In this regard, digital platforms offer a promising alternative for collecting and evaluating patient mental health data, while offering the advantage of being more easily available, adaptable, scalable and cost-effective compared to traditional, interview-based methods^16^. Existing digital mental health applications are generally considered safe^17^, although, despite their rapidly growing numbers, little evidence is available on their accuracy or efficacy^16^. Furthermore, it is anticipated that diagnostic accuracy in mental healthcare can be improved by incorporating biomarker profiling strategies, which could additionally provide a biological basis for mood disorder stratification and personalised treatment^18^.

We aimed to establish and validate a diagnostic algorithm, based on a new online mental health questionnaire and blood biomarker data, to detect BD in patients with a recent diagnosis of MDD and, hence, reduce the misdiagnosis of BD as MDD.

## Materials and methods

### Study design and participants

Data analysed here were collected as part of the Delta Study, an investigator-led study conducted by the Cambridge Centre for Neuropsychiatric Research (CCNR) at the University of Cambridge, which aimed to improve mood disorder diagnosis in participants presenting with depressive symptoms^19–22^. The primary objective of the Delta Study was to identify BD patients among patients who have recently (≤5 years)^10,11^ been diagnosed as having MDD. The study was approved by the University of Cambridge Human Biology Research Ethics Committee (approval number HBREC 2017.11) and was conducted in compliance with the Declaration of Helsinki^23^, Good Clinical Practice and ISO 14155:2011. A detailed research protocol for the Delta Study has been published previously^19^. Participants were recruited online through email, via the CCNR website and Facebook. Inclusion criteria for the study required participants to be between 18 and 45 years old, residents of the United Kingdom, at least mildly depressed (Patient Health Questionnaire-9^24^ total score ≥5), not pregnant or breastfeeding, and not suicidal. All participants read the participant information sheet and digitally provided informed consent for participation in the study. Recruitment started on 27 April 2018 and was completed on 28 September 2018. The current work complies with the Standards for Reporting of Diagnostic Accuracy Studies (STARD)^25^ and the Strengthening the Reporting of Observational Studies in Epidemiology (STROBE)^26^ guidelines.

### Procedures

Upon enrolment, participants were asked to complete a purpose-built online mental health questionnaire available through the Delta Study website. The questionnaire was developed in collaboration with experienced psychiatrists and a service user advisory group and was based on existing structured diagnostic interviews as well as a range of mental health screening questionnaires^19^. It consisted of 635 distinct questions belonging to 6 modules: (1) demographic information, (2) manic and hypomanic symptoms, (3) depressive symptoms, (4) personality traits, (5) psychiatric history and (6) other psychiatric conditions. The questionnaire was adaptive to answers given by participants, so that only relevant questions were asked, and the maximum possible number of questions asked to an individual was 382 (284 on average). Data collected from the questionnaire were used to identify participants qualifying for the study objectives, and as independent variables in statistical modelling.

Next, eligible participants who consented to providing a blood sample and completing a telephone diagnostic interview, who were free from blood-borne illnesses and had no previous diagnosis of schizophrenia, were provided with a dried blood spot (DBS) collection kit by post. The kit was designed to allow minimally invasive blood sample collection in a non-clinical setting, and was a Conformité Européenne-marked device under Article 22 of the Medical Device Regulation 2017/745. The kit included pre-injection cleaning swabs, sterile finger prick lancets, a DBS collection card (226 Spot Saver Cards, PerkinElmer), adhesive plasters and cotton pads. Detailed instructions for DBS sample collection were provided in a leaflet and as an online video. Participants were asked to spot 5 separate DBSs onto the card, after at least 6 h of fasting, and allow the card to dry for a minimum of 3 hrs at room temperature. Cards were subsequently placed in the provided resealable bags with desiccant, and returned by post using pre-paid envelopes.

The returned DBS samples were analysed for neuropsychiatric biomarker levels using a validated targeted proteomic approach^27–29^. The method targeted 203 unique peptides representing 120 proteins (Supplementary Table 1) selected based on their association with psychiatric conditions and concentration in the blood^28^. DBS samples were processed using an automated Biomek NX workstation (Beckman Coulter). Proteins were extracted from 3 mm DBS discs using 50 mM ammonium bicarbonate, followed by disulphide bond reduction with 5 mM dithiothreitol and cysteine alkylation using 10 mM iodoacetamide. Next, proteins were digested overnight with trypsin at 1:20 enzyme to protein ratio, followed by peptide purification in FNSC18 plates (Glygen Corp.) and elution with 60% acetonitrile. Stable isotope-labelled internal standard (SIS) peptides were subsequently spiked in for each target peptide to enable quantitative analysis. Infinity 1290 liquid chromatography system (Agilent) was used to separate ~3.2 µg of digested proteins on a 2.1 × 150 mm AdvanceBio Peptide Mapping column (Agilent) at 50 °C. Peptides were eluted using a gradient of acetonitrile in 0.1% formic acid from 3 to 30% over 45 minutes at 0.3 ml/min, and analysed with a triple quadrupole mass spectrometer model 6495 (Agilent) equipped with a Jet Stream ion source operated in positive ionisation mode, using dynamic multiple reaction monitoring^27^. Samples were randomised across plates, plate positions and experimental days to minimise technical bias, and quality control samples were included to monitor variation in sample preparation and instrument performance. Experimenters were blind to sample diagnostic allocation.

### Outcomes

Participants who successfully completed the online questionnaire and returned the DBS sample were invited to complete the World Health Organization World Mental Health Composite International Diagnostic Interview (CIDI), version 3.0^30^ via telephone. The CIDI is a modular diagnostic tool which is widely used in epidemiological studies on mental health^31^ and shows good concordance with structured diagnostic interviews conducted by clinicians^32^. All interviewers conducting the CIDI received in-person training from an external CIDI-certified instructor, and internal training and mentoring. Only modules of the CIDI required for the lifetime mood disorder diagnosis, i.e. the screening, depression and mania sections, were implemented. We adopted voluntary response sampling, whereby the CIDI interviews continued until pre-specified study recruitment targets were met.

### Statistical analysis

Power calculations for the study’s primary objective showed that, assuming at least 80% sensitivity of the algorithm in detecting BD previously diagnosed as MDD and at least a 20% prevalence of BD among participants recently diagnosed with MDD^8^, a minimum of 200 participants with a recent diagnosis of MDD by a medical professional were required to provide at least 80% power to detect model noninferiority against an AUROC of 0.80 at the 5% significance level. The required number of participants was increased to 300 to account for potential dropouts, as observed in previous studies. Analogous calculations demonstrated that at least 300 symptomatic participants with no baseline diagnosis of mood disorder were required for the study’s secondary objectives^19^. Additionally, we aimed to recruit 40 participants with a previous diagnosis of BD by a medical professional to validate the algorithm.

Data processing and analysis were conducted in R version 3.6.3^33^. The online mental health questionnaire data were restructured so that answers to equivalent questions were concatenated (e.g. current and past symptoms), missing values were imputed where feasible (e.g. the number of relatives with depression was set to 0 for participants with no family history of mental health conditions), and features derived from the original variables were added (guided by the design of existing diagnostic algorithms, e.g. the number of symptoms). Ordinal questionnaire data were converted to ranks, and categorical data were encoded as dummy variables. Features that were duplicated, bijections or constant were removed. All missing values in the dataset were due to the adaptive character of the questionnaire (missing not at random and more likely for MDD). Raw biomarker data were processed in Skyline version 3.1.0^34^. Peptides which were not detected (*N* = 9) were excluded from the analyses. Relative biomarker quantification was based on the ratios of abundances of the endogenous peptides over the abundances of the corresponding SIS peptides. Potential batch effects, caused by processing and analysing DBS samples across multiple plates, were adjusted for by median scaling. Biomarker level values were log_2_-transformed prior to analysis. The final number of analysed features was 1151, including 957 items from the online mental health questionnaire and 194 protein peptide measurements. The CIDI diagnosis was used as the dependent variable.

**Fig. 1:**
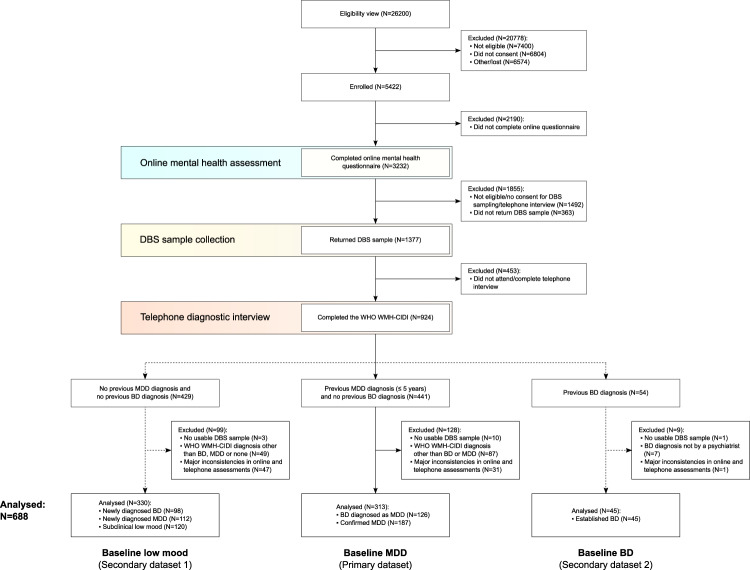
Delta Study flow diagram. The diagram shows the number of individuals who completed each step of the study and reasons for attrition. BD bipolar disorder, DBS dried blood spot, MDD major depressive disorder, WHO WMH-CIDI World Health Organization World Mental Health Composite International Diagnostic Interview.

The diagnostic algorithm was trained and validated using data from participants with a recent self-reported diagnosis of MDD, confirmed as MDD or changed to BD by the CIDI. Participants whose DBS samples were not usable, or whose answers to the screening question about elevated mood on the online questionnaire and the telephone interview were inconsistent, were excluded (Fig. 1). Extreme Gradient Boosting (XGBoost)^35^, a decision tree-based machine learning method, was selected to build the diagnostic algorithm, primarily because of its ability to handle missing values and detect non-linear relationships and interactions between variables, being robust to correlated features, as well as its interpretability. Nested cross-validation (CV), an equivalent to creating multiple train-test splits, was used to obtain robust estimates of model predictive performance in previously unseen data^36^. We used 5-fold stratified nested CV, wherein at each iteration 4 of the folds were used in the inner loop to tune model parameters and train the algorithm, and the 5th fold was used in the outer loop to test the trained model. Training of the XGBoost model was based on 5-fold stratified CV repeated three times. Tuned model parameters included the number of trees (1 to 100), tree depth (1 or 2, to allow for first order interactions) and the learning rate (0.1 or 0.3). Initial testing showed that more extensive tuning was not required. Model performance was evaluated using AUROC. To avoid overfitting, optimal model parameters were defined as those which resulted in the simplest model with AUROC within 1 standard error below the AUROC of the best performing model. The final model was fitted using the tuned parameters to all data from the inner loop, and evaluated on the test set in the outer loop. Youden’s J statistic^37^ was used to determine the optimal classification cut-off with balanced sensitivity and specificity. Nested CV was repeated 20 times, resulting in 100 models. The 95% confidence intervals (CI) were estimated for all measures of diagnostic performance as the 2.5th and 97.5th percentiles across the 100 models. Features were evaluated based on their occurrence frequency across the 100 models and mean feature importance, i.e. gain (increase in accuracy brought by a feature to the branches it occurred on). The directionality of the relationship between the predictor and outcome variables was determined using the SHapley Additive exPlanations (SHAP) method^38^. The trained models were additionally validated in symptomatic participants with no previous diagnosis of mood disorder, and in participants with a previously established diagnosis of BD. Additional analyses included training classification models using: (1) subsamples of the training data, to assess potential bias related to sample size^39^; (2) only features from the online questionnaire or only the biomarker data, to separately assess their potential utility; and (3) all available instances, i.e. including participants whose answers were inconsistent between the online and telephone assessments, to assess selection bias.

## Results

The study flow diagram is shown in Fig. 1. To achieve study recruitment targets, 5422 symptomatic individuals were enrolled, of which 3232 completed the online mental health questionnaire, 1377 provided a DBS sample and 924 completed the CIDI diagnostic interview. The average time interval between starting the online assessment and completing the CIDI interview was 14 days. Only data from participants who returned a usable DBS sample and whose answers on the online questionnaire and the telephone interview were not inconsistent were analysed (*N* = 688; Fig. 1). These included 126 participants with BD (*N* = 76 BD I and 50 BD II) diagnosed as MDD and 187 participants with confirmed MDD from the primary dataset, and 98 newly diagnosed participants with BD (*N* = 60 BD I and 38 BD II), 112 newly diagnosed participants with MDD, 120 participants with subclinical low mood (i.e. no previous mood disorder diagnosis and no mood disorder diagnosis from the CIDI), and 45 participants with a previous diagnosis of BD from the secondary datasets (Fig. 1). Demographic and clinical characteristics are shown in Table 1. Participants with BD who had been diagnosed as having MDD were, on average ± standard deviation (SD), 27.4 ± 7.2 years old, 59% female, and overweight (BMI of 28.5 ± 7.4). The mean duration of MDD diagnosis in this group was 2.7 ± 1.6 years. The majority (94%) had been treated with antidepressant medication, and the same percentage had never received any mood stabiliser treatment. Previous self-reported MDD diagnoses were made primarily by a General Practitioner (81.2%), followed by those made by a psychiatrist (18.5%) and other medical professionals (0.3%). Of the 45 participants with previously diagnosed BD, 35 (78%) reported having been initially diagnosed with MDD, with the average time between MDD and BD diagnosis in this group being 5.5 ± 5.9 years. The mean duration of existing BD diagnosis was 7.5 ± 6.8 years.

**Table 1:** Demographic and clinical characteristics of study participants.

	Baseline MDD		Baseline low mood			Baseline BD	
Diagnosis	BD	MDD	BD	MDD	Low mood	BD	*P* value
*N*	126	187	98	112	120	45	NA
Age, mean (SD), years	27.4 (7.2)	28.1 (6.9)	25.4 (5.9)	26.4 (6.2)	25.8 (6.5)	33.9 (7.8)	<0.001
Sex, *N* (%)							
Male	52 (41)	50 (27)	36 (37)	30 (27)	40 (33)	28 (62)	<0.001
Female	74 (59)	137 (73)	62 (63)	82 (73)	80 (67)	17 (38)	
BMI, mean (SD), kg/m^2^	28.5 (7.4)	28.3 (7.0)	26.3 (6.6)	26.6 (6.6)	24.6 (4.5)	28.6 (6.2)	<0.001
Ethnicity, *N* (%)							
Asian/British Asian	1 (1)	3 (2)	2 (2)	1 (1)	7 (6)	0 (0)	0.104
Black/Black British	1 (1)	0 (0)	0 (0)	0 (0)	0 (0)	0 (0)	
White	93 (74)	132 (71)	61 (62)	74 (66)	73 (61)	37 (82)	
Mixed	5 (4)	5 (3)	3 (3)	5 (4)	3 (3)	0 (0)	
Other	0 (0)	1 (1)	1 (1)	2 (2)	4 (3)	0 (0)	
Prefer not to say	0 (0)	0 (0)	0 (0)	0 (0)	0 (0)	0 (0)	
Unknown^a^	26 (21)	46 (25)	31 (32)	30 (27)	33 (28)	8 (18)	
Smoking, *N* (%)							
No	40 (32)	116 (62)	36 (37)	60 (54)	67 (56)	18 (40)	<0.001
Yes	86 (68)	71 (38)	62 (63)	52 (46)	53 (44)	27 (60)	
Alcohol consumption, *N* (%)							
No	36 (29)	45 (24)	19 (19)	16 (14)	14 (12)	16 (36)	0.001
Yes	90 (71)	142 (76)	79 (81)	96 (86)	106 (88)	29 (64)	
Recreational drug use, *N* (%)							
No	71 (56)	131 (70)	41 (42)	69 (62)	71 (59)	33 (73)	<0.001
Yes	55 (44)	56 (30)	57 (58)	43 (38)	49 (41)	12 (27)	
Education, *N* (%)							
<GCSE	3 (2)	0 (0)	4 (4)	2 (2)	1 (1)	0 (0)	0.001
GCSE	18 (14)	19 (10)	8 (8)	5 (4)	10 (8)	8 (18)	
A-level	49 (39)	46 (25)	33 (34)	23 (21)	32 (27)	12 (27)	
Undergraduate degree	37 (29)	79 (42)	33 (34)	48 (43)	57 (48)	19 (42)	
Postgraduate degree	19 (15)	43 (23)	20 (20)	34 (30)	20 (17)	6 (13)	
Employment, *N* (%)							
Employed/self-employed	81 (64)	111 (59)	62 (63)	68 (61)	68 (57)	22 (49)	<0.001
Parental leave	0 (0)	1 (1)	0 (0)	0 (0)	0 (0)	0 (0)	
Student	28 (22)	50 (27)	22 (22)	37 (33)	47 (39)	9 (20)	
Retired	0 (0)	1 (1)	0 (0)	0 (0)	0 (0)	2 (4)	
Unemployed	17 (13)	24 (13)	14 (14)	7 (6)	5 (4)	12 (27)	
Relationship status, *N* (%)							
In a relationship	80 (63)	114 (61)	57 (58)	81 (72)	81 (68)	30 (67)	0.275
Single	46 (37)	73 (39)	41 (42)	31 (28)	39 (32)	15 (33)	
Childhood trauma, *N* (%)							
No	44 (35)	84 (45)	32 (33)	55 (49)	77 (64)	15 (33)	<0.001
Yes	81 (64)	102 (55)	65 (66)	53 (47)	39 (32)	29 (64)	
Prefer not to say	1 (1)	1 (1)	1 (1)	4 (4)	4 (3)	1 (2)	
Family psychiatric history, *N* (%)							
No	27 (21)	31 (17)	27 (28)	38 (34)	41 (34)	2 (4)	<0.001
Yes	99 (79)	156 (83)	71 (72)	74 (66)	79 (66)	43 (96)	
Duration of MDD diagnosis, mean (SD), years	2.7 (1.6)	2.6 (1.5)	NA	NA	NA	5.5 (5.9)^b^	0.037
Antidepressant treatment, *N* (%)							
SSRI	116 (92)	174 (93)	27 (28)	16 (14)	14 (12)	38 (84)	<0.001
SNRI	24 (19)	22 (12)	2 (2)	2 (2)	1 (1)	20 (44)	
TCA	13 (10)	19 (10)	6 (6)	2 (2)	1 (1)	16 (36)	
Other	15 (12)	17 (9)	3 (3)	3 (3)	2 (2)	8 (18)	
None	7 (6)	9 (5)	67 (68)	91 (81)	104 (87)	5 (11)	
Duration of BD diagnosis, mean (SD), years	NA	NA	NA	NA	NA	7.5 (6.8)	NA
Mood stabiliser treatment, *N* (%)							
No	119 (94)	180 (96)	97 (99)	112 (100)	120 (100)	18 (40)	<0.001
Yes	7 (6)	7 (4)	1 (1)	0 (0)	0 (0)	27 (60)	
Psychiatric hospitalisation, *N* (%)							
No	105 (83)	170 (91)	91 (93)	111 (99)	119 (99)	25 (56)	<0.001
Yes	21 (17)	17 (9)	7 (7)	1 (1)	1 (1)	20 (44)	
PHQ-9 score, mean (SD)	16.2 (4.7)	13.8 (4.8)	14.3 (4.7)	12.9 (4.7)	9.9 (3.7)	13.1 (4.4)	<0.001
WEMWBS score, mean (SD)	33.0 (7.5)	35.1 (7.3)	35.2 (7.4)	36.8 (7.3)	42.0 (7.0)	36.5 (6.7)	<0.001
Fasting at DBS collection							
No	2 (2)	1 (1)	4 (4)	0 (0)	2 (2)	2 (4)	0.097
Yes	124 (98)	186 (99)	94 (96)	112 (100)	118 (98)	43 (96)	

*P* values were obtained from the Kruskal–Wallis test for continuous variables and χ^2^ test for categorical variables.

*BD* bipolar disorder, *BMI* body mass index, *DBS* dried blood spot, *GCSC* General Certificate of Secondary Education, *MDD* major depressive disorder, *NA* not applicable, *PHQ-9* Patient Health Questionnaire-9, *SD* standard deviation, *SNRI* serotonin-norepinephrine reuptake inhibitor, *SSRI* selective serotonin reuptake inhibitor, *TCA* tricyclic antidepressant. *WEMWBS* Warwick-Edinburgh Mental Wellbeing Scale.

^a^Information on ethnicity was collected at 6 months follow-up, which was not completed by all participants.

^b^Until BD diagnosis (*N* = 35).

**Fig. 2:**
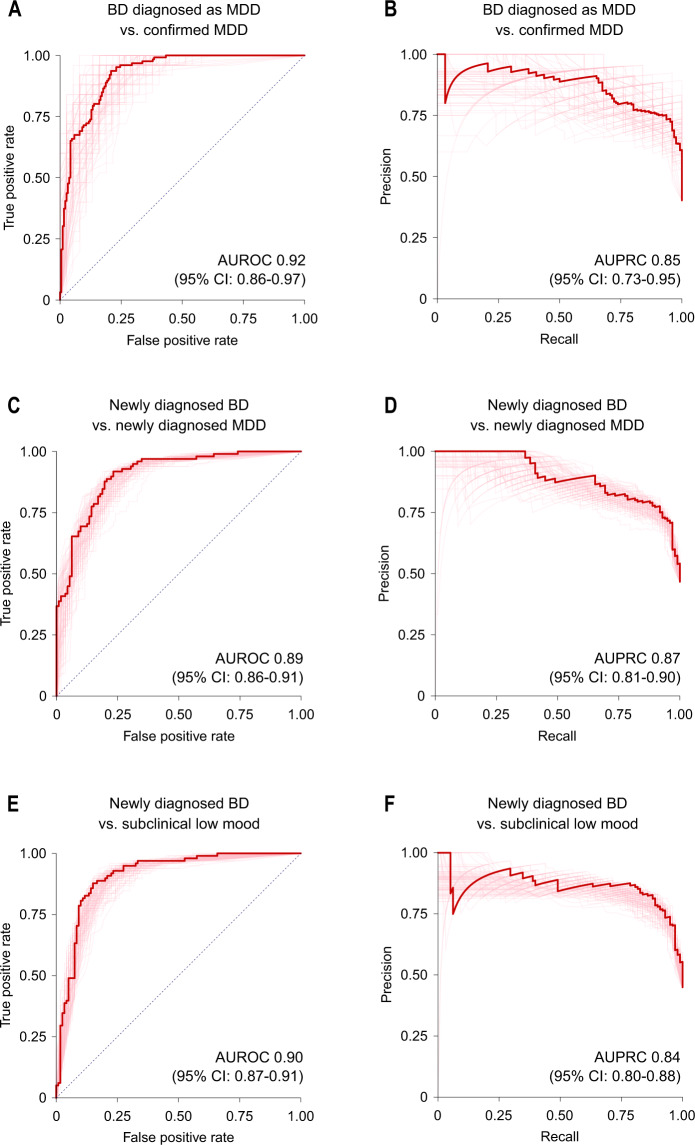
Receiver operating characteristic and precision-recall curves for prediction of bipolar disorder diagnosis. **A**, **B** Out-of-fold results of nested cross-validation in the primary dataset (*N* = 126 BD previously diagnosed as MDD vs. N = 187 confirmed MDD). Thick lines represent curves calculated from probabilities averaged across all models. **C**–**F** Validation in baseline low mood group (*N* = 98 newly diagnosed BD vs. N = 112 newly diagnosed MDD (**C**, **D**) and vs. *N* = 120 with subclinical depressive symptoms (**E**, **F**)). AUROC and AUPRC values represent mean (95% CI). AUPRC area under the precision-recall curve, AUROC area under the receiver operating characteristic curve, BD bipolar disorder, CI confidence intervals, MDD major depressive disorder.

The trained algorithms showed an out-of-fold AUROC of 0.92 (95% CI: 0.86–0.97) in separating participants with BD previously diagnosed as MDD from those with confirmed MDD (Fig. 2A). In subgroup analyses, the AUROC was higher in participants with BD I (0.94; 0.88–0.98) than in participants with BD II (0.88; 0.78–0.95). The out-of-fold area under the precision-recall curve (AUPRC) in the primary dataset was 0.85 (0.73–0.95; Fig. 2B). Detailed estimates of out-of-fold model performance are summarised in Table 2.

**Table 2:** Out-of-fold model performance in the primary and secondary datasets.

	Baseline MDD	Baseline low mood		Baseline BD
	126 BD vs. 187 MDD	98 BD vs. 112 MDD	98 BD vs. 120 low mood	45 BD
AUROC	0.92 (0.86–0.97)	0.89 (0.86–0.91)	0.90 (0.87–0.91)	NA
AUPRC	0.85 (0.73–0.95)	0.87 (0.81–0.90)	0.84 (0.80–0.88)	NA
Accuracy	0.83 (0.76–0.91)	0.80 (0.75–0.84)	0.82 (0.77–0.86)	NA
Sensitivity	0.84 (0.66–1.00)	0.77 (0.61–0.90)	0.77 (0.61–0.90)	0.86 (0.57–0.96)
Specificity	0.83 (0.65–0.95)	0.83 (0.76–0.92)	0.86 (0.77–0.94)	NA
PPV	0.77 (0.64–0.91)	0.80 (0.76–0.88)	0.83 (0.76–0.89)	NA
NPV	0.89 (0.79–1.00)	0.81 (0.72–0.90)	0.83 (0.74–0.91)	NA

Values are shown as mean (95% confidence intervals).

*AUPRC* area under the precision-recall curve, *AUROC* area under the receiver operating characteristic curve, *BD* bipolar disorder, *MDD* major depressive disorder, *NA* not applicable, *NPV* negative predictive value, *PPV* positive predictive value.

The median number of features across the models was 9, with the interquartile range between 6 and 15. Performance was driven primarily by the 5 features present in the majority of the models, namely elevated mood, grandiose delusions, talkativeness, recklessness, and risky behaviour (Table 3). Among the 30 most frequently selected features, 26 were from the online questionnaire, including questions on mania/hypomania, emotional instability, psychiatric history and comorbidities, and quality of life, and four were biomarker measurements. Directionality of the relationships is shown in Supplementary Fig. 1. Follow-up analyses showed that models built using the online questionnaire or blood biomarker data separately had respective AUROCs of 0.92 (95% CI: 0.85–0.97) and 0.50 (0.34–0.62). Details of the models trained using only the online questionnaire data or the biomarker data are shown is Supplementary Tables 2–5.

**Table 3:** Top predictors of bipolar disorder.

Feature	Frequency	Importance, mean (SD)	Category
Elevated mood	0.99	0.182 (0.080)	Bipolar/hypomania
Grandiosity	0.87	0.095 (0.042)	Bipolar/hypomania
More talkative	0.82	0.193 (0.089)	Bipolar/hypomania
Recklessness (HMQ)	0.82	0.122 (0.087)	Bipolar/hypomania
Others: risky behaviour	0.77	0.232 (0.122)	Bipolar/hypomania
Others: speaking faster	0.38	0.033 (0.018)	Bipolar/hypomania
Recklessness (PQ)	0.38	0.041 (0.016)	Emotional instability
Increased energy	0.37	0.055 (0.030)	Bipolar/hypomania
Mood lability	0.35	0.037 (0.013)	Emotional instability
Risky behaviour	0.31	0.225 (0.125)	Bipolar/hypomania
Episode duration	0.30	0.050 (0.021)	Bipolar/hypomania
≥3 symptoms	0.30	0.143 (0.099)	Bipolar/hypomania
Second-degree relatives with MDD	0.27	0.030 (0.014)	History/comorbidities
Past elevated mood	0.25	0.025 (0.009)	Bipolar/hypomania
Fear of abandonment	0.22	0.031 (0.013)	Emotional instability
Number of episodes	0.21	0.050 (0.021)	Bipolar/hypomania
Self-image instability	0.18	0.034 (0.014)	Emotional instability
Racing thoughts	0.14	0.065 (0.045)	Bipolar/hypomania
Feeling loved (2 weeks)	0.14	0.022 (0.008)	Quality of life
KNG1 (YFIDFVAR)	0.12	0.022 (0.008)	Biomarker
Unstable relationships	0.12	0.023 (0.006)	Emotional instability
IGHG1 (FNWYVDGVEVHNAK)	0.11	0.014 (0.005)	Biomarker
Recklessness (BDQ)	0.10	0.040 (0.016)	Bipolar/hypomania
Functional impairment/Hospitalisation	0.09	0.055 (0.031)	Bipolar/hypomania
TSP1 (GTLLALER)	0.09	0.015 (0.007)	Biomarker
Social activity	0.08	0.031 (0.023)	Bipolar/hypomania
APOA1 (ATEHLSTLSEK)	0.07	0.008 (0.008)	Biomarker
Productivity	0.07	0.017 (0.008)	Bipolar/hypomania
Sleep distress	0.07	0.004 (0.003)	History/comorbidities
Duration of social phobia	0.07	0.010 (0.004)	History/comorbidities

Table shows 30 most frequently selected features and their importance (i.e. gain). Biomarkers are labelled as ‘UniProtKB protein ID (target peptide sequence)’.

*APOA1* apolipoprotein A1, *BDQ* bipolar disorder questionnaire, *HMQ* hypomania questionnaire, *IGHG1* immunoglobulin heavy constant gamma 1, *KNG1* kininogen 1, *MDD* major depressive disorder, *PQ* personality disorder questionnaire, *SD* standard deviation, *TSP1* thrombospondin-1, *UniProtKB* UniProt Knowledgebase.

Additional validation in the secondary datasets showed that the models separated participants with newly diagnosed BD from those with newly diagnosed MDD and subclinical low mood with respective AUROCs of 0.89 (95% CI: 0.86–0.91) and 0.90 (0.87–0.91), and AUPRCs of 0.87 (0.81–0.90) and 0.84 (0.80–0.88; Fig. 2C–F and Table 2). Furthermore, the models predicted the correct diagnosis in 86% (57–96%) of participants with an established diagnosis of BD (Table 2).

Sensitivity analyses showed that the study was sufficiently powered, as indicated by the plateauing model performance at training set sizes ≥100 (Supplementary Figs. 2 and 3). An additional analysis indicated that some selection bias might have been introduced when excluding participants who gave inconsistent answers on the online and telephone assessments. Including those participants in the analysis returned a test AUROC of 0.86 (95% CI: 0.78–0.93) when distinguishing participants with BD previously diagnosed as MDD from those with confirmed MDD.

## Discussion

The main aim of the present study was to develop a diagnostic algorithm, based on an online mental health questionnaire and blood biomarker data, to identify BD patients among recently diagnosed MDD patients. The trained models achieved an average test AUROC of 0.92, with a mean accuracy of 0.83, representing a 38% improvement compared to the baseline accuracy of 0.60, i.e. the proportion of correctly diagnosed patients with MDD in the primary dataset (187/313). While we pre-specified the AUROC threshold for clinical relevance at 0.80^19^, the obtained estimate of above 0.90 is considered ‘excellent’^40^ or ‘almost perfect’^32^ for mental health disorder diagnosis. The remaining discrepancy between the algorithm and the CIDI outcomes is not unexpected given general diagnostic uncertainty surrounding psychiatric conditions, whereby even the ‘gold standard’ measures disagree in a small number of cases^41^.

The present results confirmed self-reported elevated mood, grandiosity, talkativeness, and recklessness as core features of BD. Therefore, it is feasible that a simple, low-cost and highly scalable digital self-reporting tool could help expedite a correct diagnosis of BD by early capturing of emerging symptoms in patients presenting with depressive symptoms. The adaptive design of the questionnaire could be further streamlined by applying iterative machine learning algorithms, such as Bayesian updating or reinforcement learning, to offer dynamic question selection personalised to individual users. However, this approach would require a substantially larger training set size and limit the amount of user data available for future exploratory analyses. Introducing such digital instruments into primary healthcare, where resources are scarce and where symptoms of BD often remain undiagnosed^42^, has the potential to lessen the burden experienced by both patients and medical professionals, and therefore reduce the overall load on the healthcare system. In particular, such an approach could constitute a cost- and time-effective alternative to conventional, interview-based methods, while allowing for a more comprehensive symptom assessment and identification of patients who require specialty care services early in the mental health triage process.

In addition, among the top ranked predictors were more objective features such as symptoms reported as being ‘observed by others’ concerning risky behaviour and speaking faster, the number of second-degree relatives with MDD, sleep disturbances, and several biomarkers including kininogen-1 (KNG1) and thrombospondin-1 (TSP1), proteins previously reported to discriminate BD from MDD^43,44^. Although biomarker data alone were not predictive of the disease status, their selection alongside digital features in some models suggests their potential utility in subgroups of patients or in specific symptom contexts. All together, these results indicate the emerging potential for more objective and systematic diagnostic approaches, such as digital phenotyping of symptoms^45^, multi-reporter assessment systems^46^, and genetic^47^ and proteomic^44,48^ biomarker profiling, in aiding the diagnosis of BD.

This study has a number of advantages compared to previous studies aiming to distinguish BD from MDD during depressive episodes. To our knowledge it is the largest investigation, with more than twice as many participants as the largest study to date (*N* = 313 vs. *N* = 112)^44^. It has also been more extensively validated, through the application of nested CV in the primary dataset and additional testing in two secondary datasets, while previous studies employed 10-fold or leave-one-out CV and no external test sets. Although not directly comparable, the algorithm outperformed existing models, for which the maximum AUROC was 0.9058^49^. The current study is also unique in its aim to develop a robust heuristic algorithm to detect BD in individuals with a recent diagnosis of MDD based on combined symptom and biomarker data.

The present results should be interpreted within their limitations. Due to recruiting participants through the internet, and in order to meet specific study recruitment targets, the analysed population might be biased and not representative of patients presenting in primary or secondary care services. In addition, we did not have access to participants’ medical records and could not verify the self-reported psychiatric history. Also, despite attempting to control for the consistency of answers between the online and telephone assessments, other inaccuracies might have remained in the dataset. It is also important to note that such step would be unfeasible in real-life applications, and alternative approaches, built into the online questionnaire, should be employed to protect data integrity^50^. Finally, BD might have not been detected in participants who have not yet experienced a manic/hypomanic episode, and a longitudinal study would be required to determine the correct diagnosis for those individuals.

In conclusion, our study provides a proof of concept that an evidence-based algorithm can accurately detect BD in patients recently diagnosed with MDD. The results may generalise to other clinically relevant populations. Further work is required to rigorously assess the potential of incorporating such algorithms into primary healthcare, where the majority of MDD diagnoses are made, to expedite the diagnosis of BD and reduce workload for healthcare professionals.

## Supplementary information

Supplementary Material

## Data Availability

Upon request from the corresponding author SB.
