# Supplementary material for: A machine learning algorithm to differentiate bipolar disorder from major depressive disorder using an online mental health questionnaire and blood biomarker data
Source: Transl Psychiatry. 2021 Jan 12;11:41. doi: 10.1038/s41398-020-01181-x (PMC7804187; doi:10.1038/s41398-020-01181-x)
Supplement: Supplementary file 1 — Supplementary Material [file 41398_2020_1181_MOESM1_ESM.docx]

**SUPPLEMENTARY MATERIAL**

**Supplementary Table 1**

| **Protein No.** | **UniProtKB entry** | **UniProtKB accession** | **Peptide No.** | **Peptide sequence** | **QC** |
| --- | --- | --- | --- | --- | --- |
| 1 | A1AG1_HUMAN | P02763 | 1 | SDVVYTDWK |  |
| 2 | A1AG2_HUMAN | P19652 | 2 | EHVAHLLFLR | fail |
| 3 | A1AT_HUMAN | P01009 | 3 | LSITGTYDLK |  |
| 3 | A1AT_HUMAN | P01009 | 4 | SPLFMGK |  |
| 3 | A1AT_HUMAN | P01009 | 5 | SVLGQLGITK |  |
| 4 | A1BG_HUMAN | P04217 | 6 | ATWSGAVLAGR |  |
| 4 | A1BG_HUMAN | P04217 | 7 | GVTFLLR |  |
| 4 | A1BG_HUMAN | P04217 | 8 | LLELTGPK |  |
| 4 | A1BG_HUMAN | P04217 | 9 | SGLSTGWTQLSK |  |
| 5 | A2AP_HUMAN | P08697 | 10 | DFLQSLK |  |
| 5 | A2AP_HUMAN | P08697 | 11 | FDPSLTQR |  |
| 5 | A2AP_HUMAN | P08697 | 12 | LFGPDLK |  |
| 6 | A2GL_HUMAN | P02750 | 13 | VAAGAFQGLR |  |
| 7 | A2MG_HUMAN | P01023 | 14 | AIGYLNTGYQR |  |
| 7 | A2MG_HUMAN | P01023 | 15 | NEDSLVFVQTDK |  |
| 8 | AACT_HUMAN | P01011 | 16 | ADLSGITGAR |  |
| 8 | AACT_HUMAN | P01011 | 17 | EIGELYLPK |  |
| 8 | AACT_HUMAN | P01011 | 18 | EQLSLLDR |  |
| 9 | AL1A1_HUMAN | P00352 | 19 | ILDLIESGK |  |
| 10 | ALBU_HUMAN | P02768 | 20 | AAFTECCQAADK |  |
| 10 | ALBU_HUMAN | P02768 | 21 | ETYGEMADCCAK |  |
| 10 | ALBU_HUMAN | P02768 | 22 | QNCELFEQLGEYK |  |
| 10 | ALBU_HUMAN | P02768 | 23 | YLYEIAR |  |
| 11 | ALDOA_HUMAN | P04075 | 24 | ALANSLACQGK |  |
| 11 | ALDOA_HUMAN | P04075 | 25 | QLLLTADDR |  |
| 12 | AMBP_HUMAN | P02760 | 26 | ETLLQDFR |  |
| 12 | AMBP_HUMAN | P02760 | 27 | TVAACNLPIVR |  |
| 13 | ANGT_HUMAN | P01019 | 28 | ALQDQLVLVAAK |  |
| 13 | ANGT_HUMAN | P01019 | 29 | SLDFTELDVAAEK |  |
| 14 | ANT3_HUMAN | P01008 | 30 | EVPLNTIIFMGR |  |
| 14 | ANT3_HUMAN | P01008 | 31 | FATTFYQHLADSK |  |
| 14 | ANT3_HUMAN | P01008 | 32 | FDTISEK |  |
| 14 | ANT3_HUMAN | P01008 | 33 | LPGIVAEGR |  |
| 15 | APOA1_HUMAN | P02647 | 34 | ATEHLSTLSEK |  |
| 15 | APOA1_HUMAN | P02647 | 35 | LLDNWDSVTSTFSK |  |
| 15 | APOA1_HUMAN | P02647 | 36 | VSFLSALEEYTK |  |
| 16 | APOA2_HUMAN | P02652 | 37 | EQLTPLIK |  |
| 16 | APOA2_HUMAN | P02652 | 38 | SPELQAEAK |  |
| 17 | APOA4_HUMAN | P06727 | 39 | ALVQQMEQLR |  |
| 17 | APOA4_HUMAN | P06727 | 40 | IDQNVEELK |  |
| 17 | APOA4_HUMAN | P06727 | 41 | ISASAEELR |  |
| 17 | APOA4_HUMAN | P06727 | 42 | LAPLAEDVR |  |
| 17 | APOA4_HUMAN | P06727 | 43 | LLPHANEVSQK |  |
| 18 | APOB_HUMAN | P04114 | 44 | TGISPLALIK |  |
| 19 | APOC1_HUMAN | P02654 | 45 | EFGNTLEDK |  |
| 19 | APOC1_HUMAN | P02654 | 46 | EWFSETFQK |  |
| 20 | APOC2_HUMAN | P02655 | 47 | TAAQNLYEK |  |
| 20 | APOC2_HUMAN | P02655 | 48 | TYLPAVDEK |  |
| 21 | APOC3_HUMAN | P02656 | 49 | GWVTDGFSSLK |  |
| 22 | APOD_HUMAN | P05090 | 50 | NILTSNNIDVK |  |
| 22 | APOD_HUMAN | P05090 | 51 | VLNQELR |  |
| 23 | APOE_HUMAN | P02649 | 52 | AATVGSLAGQPLQER |  |
| 23 | APOE_HUMAN | P02649 | 53 | ALMDETMK |  |
| 23 | APOE_HUMAN | P02649 | 54 | LGPLVEQGR |  |
| 23 | APOE_HUMAN | P02649 | 55 | SELEEQLTPVAEETR |  |
| 24 | APOH_HUMAN | P02749 | 56 | EHSSLAFWK |  |
| 25 | APOL1_HUMAN | O14791 | 57 | LNILNNNYK |  |
| 25 | APOL1_HUMAN | O14791 | 58 | VNEPSILEMSR |  |
| 26 | APOM_HUMAN | O95445 | 59 | AFLLTPR | fail |
| 26 | APOM_HUMAN | O95445 | 60 | FLLYNR |  |
| 26 | APOM_HUMAN | O95445 | 61 | SLTSCLDSK |  |
| 27 | C1QB_HUMAN | P02746 | 62 | GNLCVNLMR |  |
| 28 | C1QC_HUMAN | P02747 | 63 | FQSVFTVTR |  |
| 28 | C1QC_HUMAN | P02747 | 64 | TNQVNSGGVLLR |  |
| 29 | C1R_HUMAN | P00736 | 65 | YTTEIIK |  |
| 30 | C1RL_HUMAN | Q9NZP8 | 66 | VVVHPDYR |  |
| 31 | C1S_HUMAN | P09871 | 67 | LLEVPEGR |  |
| 32 | C4BPA_HUMAN | P04003 | 68 | EDVYVVGTVLR |  |
| 32 | C4BPA_HUMAN | P04003 | 69 | GYILVGQAK |  |
| 32 | C4BPA_HUMAN | P04003 | 70 | YTCLPGYVR |  |
| 33 | CAH1_HUMAN | P00915 | 71 | ADGLAVIGVLMK |  |
| 34 | CAH2_HUMAN | P00918 | 72 | GGPLDGTYR |  |
| 34 | CAH2_HUMAN | P00918 | 73 | SADFTNFDPR |  |
| 34 | CAH2_HUMAN | P00918 | 74 | VVDVLDSIK |  |
| 35 | CATA_HUMAN | P04040 | 75 | LNVITVGPR |  |
| 36 | CBG_HUMAN | P08185 | 76 | GTWTQPFDLASTR |  |
| 37 | CD44_HUMAN | P16070 | 77 | YGFIEGHVVIPR |  |
| 38 | CD5L_HUMAN | O43866 | 78 | IWLDNVR |  |
| 38 | CD5L_HUMAN | O43866 | 79 | LVGGLHR |  |
| 39 | CERU_HUMAN | P00450 | 80 | DIASGLIGPLIICK |  |
| 39 | CERU_HUMAN | P00450 | 81 | EYTDASFTNR |  |
| 40 | CFAB_HUMAN | P00751 | 82 | DISEVVTPR |  |
| 40 | CFAB_HUMAN | P00751 | 83 | DLLYIGK |  |
| 40 | CFAB_HUMAN | P00751 | 84 | EELLPAQDIK |  |
| 40 | CFAB_HUMAN | P00751 | 85 | YGLVTYATYPK |  |
| 41 | CFAH_HUMAN | P08603 | 86 | IDVHLVPDR |  |
| 41 | CFAH_HUMAN | P08603 | 87 | NGFYPATR |  |
| 42 | CFAI_HUMAN | P05156 | 88 | IVIEYVDR |  |
| 43 | CHSP1_HUMAN | Q9Y2V2 | 89 | LQAVEVVITHLAPGTK | fail |
| 44 | CLUS_HUMAN | P10909 | 90 | ASSIIDELFQDR |  |
| 44 | CLUS_HUMAN | P10909 | 91 | ELDESLQVAER |  |
| 44 | CLUS_HUMAN | P10909 | 92 | FMETVAEK |  |
| 44 | CLUS_HUMAN | P10909 | 93 | IDSLLENDR |  |
| 45 | CO3_HUMAN | P01024 | 94 | AGDFLEANYMNLQR |  |
| 45 | CO3_HUMAN | P01024 | 95 | GYTQQLAFR |  |
| 45 | CO3_HUMAN | P01024 | 96 | SGIPIVTSPYQIHFTK | fail |
| 45 | CO3_HUMAN | P01024 | 97 | TGLQEVEVK |  |
| 46 | CO4A_HUMAN | P0C0L4 | 98 | DFALLSLQVPLK |  |
| 46 | CO4A_HUMAN | P0C0L4 | 99 | VGDTLNLNLR |  |
| 46 | CO4A_HUMAN | P0C0L4 | 100 | VLSLAQEQVGGSPEK |  |
| 47 | CO8A_HUMAN | P07357 | 101 | AMAVEDIISR |  |
| 47 | CO8A_HUMAN | P07357 | 102 | HTSLGPLEAK |  |
| 48 | CO8B_HUMAN | P07358 | 103 | SGFSFGFK |  |
| 49 | CO9_HUMAN | P02748 | 104 | LSPIYNLVPVK |  |
| 49 | CO9_HUMAN | P02748 | 105 | VVEESELAR |  |
| 50 | CXCL7_HUMAN | P02775 | 106 | NIQSLEVIGK |  |
| 51 | DEMA_HUMAN | Q08495 | 107 | VTSNLGK |  |
| 52 | ENOA_HUMAN | P06733 | 108 | TIAPALVSK |  |
| 53 | F13A_HUMAN | P00488 | 109 | STVLTIPEIIIK |  |
| 54 | FA12_HUMAN | P00748 | 110 | VVGGLVALR | fail |
| 55 | FETUB_HUMAN | Q9UGM5 | 111 | LVVLPFPK |  |
| 56 | FIBA_HUMAN | P02671 | 112 | GSESGIFTNTK |  |
| 57 | FIBB_HUMAN | P02675 | 113 | AHYGGFTVQNEANK |  |
| 58 | FIBG_HUMAN | P02679 | 114 | EGFGHLSPTGTTEFWLGNEK |  |
| 58 | FIBG_HUMAN | P02679 | 115 | IHLISTQSAIPYALR | fail |
| 59 | FINC_HUMAN | P02751 | 116 | SYTITGLQPGTDYK |  |
| 59 | FINC_HUMAN | P02751 | 117 | YSFCTDHTVLVQTR |  |
| 60 | GDIB_HUMAN | P50395 | 118 | DLGTESQIFISR |  |
| 60 | GDIB_HUMAN | P50395 | 119 | FVSISDLLVPK |  |
| 61 | GELS_HUMAN | P06396 | 120 | AGALNSNDAFVLK |  |
| 61 | GELS_HUMAN | P06396 | 121 | SEDCFILDHGK |  |
| 62 | H4_HUMAN | P62805 | 122 | DAVTYTEHAK |  |
| 62 | H4_HUMAN | P62805 | 123 | VFLENVIR |  |
| 63 | HABP2_HUMAN | Q14520 | 124 | VVLGDQDLK |  |
| 64 | HBD_HUMAN | P02042 | 125 | LLGNVLVCVLAR | fail |
| 65 | HBG1_HUMAN | P69891 | 126 | MVTAVASALSSR |  |
| 66 | HEMO_HUMAN | P02790 | 127 | NFPSPVDAAFR |  |
| 66 | HEMO_HUMAN | P02790 | 128 | VDGALCMEK |  |
| 67 | HEP2_HUMAN | P05546 | 129 | FAFNLYR |  |
| 67 | HEP2_HUMAN | P05546 | 130 | IAIDLFK |  |
| 67 | HEP2_HUMAN | P05546 | 131 | TLEAQLTPR |  |
| 68 | HINT1_HUMAN | P49773 | 132 | IIFEDDR |  |
| 69 | HPT_HUMAN | P00738 | 133 | DYAEVGR |  |
| 69 | HPT_HUMAN | P00738 | 134 | VGYVSGWGR |  |
| 69 | HPT_HUMAN | P00738 | 135 | VTSIQDWVQK |  |
| 70 | HRG_HUMAN | P04196 | 136 | GGEGTGYFVDFSVR |  |
| 71 | IC1_HUMAN | P05155 | 137 | FQPTLLTLPR |  |
| 71 | IC1_HUMAN | P05155 | 138 | LLDSLPSDTR |  |
| 71 | IC1_HUMAN | P05155 | 139 | TNLESILSYPK |  |
| 72 | IF4B_HUMAN | P23588 | 140 | SILPTAPR |  |
| 73 | IGHA1_HUMAN | P01876 | 141 | TPLTATLSK |  |
| 74 | IGHG1_HUMAN | P01857 | 142 | FNWYVDGVEVHNAK |  |
| 74 | IGHG1_HUMAN | P01857 | 143 | GPSVFPLAPSSK |  |
| 75 | IGHG2_HUMAN | P01859 | 144 | GLPAPIEK | fail |
| 76 | IGHG3_HUMAN | P01860 | 145 | DTLMISR |  |
| 76 | IGHG3_HUMAN | P01860 | 146 | NQVSLTCLVK |  |
| 77 | IGHM_HUMAN | P01871 | 147 | GFPSVLR |  |
| 77 | IGHM_HUMAN | P01871 | 148 | QIQVSWLR |  |
| 77 | IGHM_HUMAN | P01871 | 149 | YAATSQVLLPSK |  |
| 78 | ITIH1_HUMAN | P19827 | 150 | AAISGENAGLVR |  |
| 79 | ITIH4_HUMAN | Q14624 | 151 | GPDVLTATVSGK |  |
| 79 | ITIH4_HUMAN | Q14624 | 152 | ILDDLSPR |  |
| 80 | KAD1_HUMAN | P00568 | 153 | IIFVVGGPGSGK |  |
| 81 | KNG1_HUMAN | P01042 | 154 | DIPTNSPELEETLTHTITK |  |
| 81 | KNG1_HUMAN | P01042 | 155 | TVGSDTFYSFK |  |
| 81 | KNG1_HUMAN | P01042 | 156 | YFIDFVAR |  |
| 82 | KPYR_HUMAN | P30613 | 157 | GDLGIEIPAEK |  |
| 83 | LEG3_HUMAN | P17931 | 158 | IALDFQR |  |
| 84 | LUM_HUMAN | P51884 | 159 | SLEDLQLTHNK |  |
| 85 | MUCB_HUMAN | P04220 | 160 | GQPLSPEK |  |
| 86 | NDKA_HUMAN | P15531 | 161 | DRPFFAGLVK |  |
| 87 | PARK7_HUMAN | Q99497 | 162 | ALVILAK |  |
| 87 | PARK7_HUMAN | Q99497 | 163 | DGLILTSR |  |
| 88 | PEBP1_HUMAN | P30086 | 164 | LYEQLSGK |  |
| 89 | PEDF_HUMAN | P36955 | 165 | ELLDTVTAPQK |  |
| 89 | PEDF_HUMAN | P36955 | 166 | TVQAVLTVPK |  |
| 90 | PERM_HUMAN | P05164 | 167 | IANVFTNAFR |  |
| 91 | PLMN_HUMAN | P00747 | 168 | FVTWIEGVMR |  |
| 91 | PLMN_HUMAN | P00747 | 169 | HSIFTPETNPR | fail |
| 92 | PMGE_HUMAN | P07738 | 170 | HYGALIGLNR |  |
| 93 | PNPH_HUMAN | P00491 | 171 | FEVGDIMLIR |  |
| 93 | PNPH_HUMAN | P00491 | 172 | VFGFSLITNK |  |
| 94 | PON1_HUMAN | P27169 | 173 | LLIGTVFHK |  |
| 95 | PPAC_HUMAN | P24666 | 174 | IELLGSYDPQK |  |
| 96 | PPIA_HUMAN | P62937 | 175 | FEDENFILK |  |
| 97 | PRDX1_HUMAN | Q06830 | 176 | ADEGISFR |  |
| 98 | PRDX2_HUMAN | P32119 | 177 | SVDEALR |  |
| 98 | PRDX2_HUMAN | P32119 | 178 | TDEGIAYR |  |
| 99 | PRDX6_HUMAN | P30041 | 179 | LSILYPATTGR |  |
| 100 | PROF1_HUMAN | P07737 | 180 | TLVLLMGK |  |
| 101 | PRS6A_HUMAN | P17980 | 181 | DAFALAK |  |
| 102 | PRS8_HUMAN | P62195 | 182 | FIGEGAR |  |
| 103 | PSA2_HUMAN | P25787 | 183 | AANGVVLATEK |  |
| 104 | PSA6_HUMAN | P60900 | 184 | HITIFSPEGR |  |
| 105 | PSB7_HUMAN | Q99436 | 185 | GTTAVLTEK |  |
| 106 | RANG_HUMAN | P43487 | 186 | FLNAENAQK |  |
| 107 | SAA4_HUMAN | P35542 | 187 | EALQGVGDMGR |  |
| 108 | SAMP_HUMAN | P02743 | 188 | IVLGQEQDSYGGK |  |
| 109 | SH3L3_HUMAN | Q9H299 | 189 | VYSTSVTGSR |  |
| 110 | TBA4A_HUMAN | P68366 | 190 | EIIDPVLDR |  |
| 111 | THBG_HUMAN | P05543 | 191 | NALALFVLPK |  |
| 112 | THIO_HUMAN | P10599 | 192 | VGEFSGANK |  |
| 113 | THRB_HUMAN | P00734 | 193 | ELLESYIDGR |  |
| 114 | TPIS_HUMAN | P60174 | 194 | FFVGGNWK |  |
| 115 | TRFE_HUMAN | P02787 | 195 | EGYYGYTGAFR |  |
| 115 | TRFE_HUMAN | P02787 | 196 | MYLGYEYVTAIR |  |
| 116 | TSP1_HUMAN | P07996 | 197 | GTLLALER |  |
| 117 | UB2L3_HUMAN | P68036 | 198 | IYHPNIDEK |  |
| 118 | VTDB_HUMAN | P02774 | 199 | HLSLLTTLSNR |  |
| 118 | VTDB_HUMAN | P02774 | 200 | VLEPTLK |  |
| 119 | VTNC_HUMAN | P04004 | 201 | DVWGIEGPIDAAFTR |  |
| 119 | VTNC_HUMAN | P04004 | 202 | DWHGVPGQVDAAMAGR |  |
| 120 | WDR1_HUMAN | O75083 | 203 | VFASLPQVER |  |

**Supplementary Table 1. List of proteins and peptides measured using selective reaction monitoring mass spectrometry.** UniProtKB=UniProt Knowledgebase. QC=quality control information.

|  | **Baseline MDD** | **Baseline low mood** | | **Baseline BD** |
| --- | --- | --- | --- | --- |
|  | **126 BD vs. 187 MDD** | **98 BD vs. 112 MDD** | **98 BD vs. 120 low mood** | **45 BD** |
| **AUROC** | 0.92 (0.85-0.97) | 0.89 (0.86-0.91) | 0.90 (0.88-0.92) | NA |
| **AUPRC** | 0.86 (0.73-0.96) | 0.86 (0.82-0.90) | 0.85 (0.81-0.88) | NA |
| **Accuracy** | 0.83 (0.75-0.94) | 0.80 (0.75-0.85) | 0.82 (0.78-0.86) | NA |
| **Sensitivity** | 0.84 (0.64-0.96) | 0.75 (0.61-0.89) | 0.75 (0.61-0.89) | 0.85 (0.63-0.96) |
| **Specificity** | 0.83 (0.69-0.95) | 0.84 (0.76-0.90) | 0.88 (0.80-0.94) | NA |
| **PPV** | 0.78 (0.65-0.91) | 0.80 (0.76-0.85) | 0.84 (0.78-0.89) | NA |
| **NPV** | 0.89 (0.80-0.97) | 0.80 (0.71-0.89) | 0.82 (0.74-0.90) | NA |

**Supplementary Table 2. Out-of-fold performance of models based on the online questionnaire data.** Values are shown as mean (95% confidence intervals). AUPRC=area under the precision-recall curve. AUROC=area under the receiver operating characteristic curve. BD=bipolar disorder. MDD=major depressive disorder. NA=not applicable. NPV=negative predictive value. PPV=positive predictive value.

| **Feature** | **Frequency** | **Importance, mean (SD)** | **Category** |
| --- | --- | --- | --- |
| Elevated mood | 0.99 | 0.161 (0.078) | Bipolar/hypomania |
| Grandiosity | 0.91 | 0.092 (0.036) | Bipolar/hypomania |
| Recklessness (HMQ) | 0.91 | 0.122 (0.080) | Bipolar/hypomania |
| More talkative | 0.87 | 0.184 (0.105) | Bipolar/hypomania |
| Others: risky behaviour | 0.77 | 0.199 (0.117) | Bipolar/hypomania |
| Others: speaking faster | 0.55 | 0.033 (0.014) | Bipolar/hypomania |
| Increased energy | 0.51 | 0.045 (0.030) | Bipolar/hypomania |
| Mood lability | 0.50 | 0.034 (0.017) | Emotional instability |
| Recklessness (PQ) | 0.50 | 0.032 (0.018) | Emotional instability |
| Episode duration | 0.44 | 0.038 (0.020) | Bipolar/hypomania |
| Fear of abandonment | 0.38 | 0.030 (0.013) | Emotional instability |
| Past elevated mood | 0.35 | 0.021 (0.008) | Bipolar/hypomania |
| Second-degree relatives with MDD | 0.34 | 0.027 (0.012) | History/comorbidities |
| Self-image instability | 0.33 | 0.028 (0.011) | Emotional instability |
| Number of episodes | 0.28 | 0.053 (0.023) | Bipolar/hypomania |
| Risky behaviour | 0.28 | 0.208 (0.095) | Bipolar/hypomania |
| ≥3 symptoms | 0.25 | 0.132 (0.086) | Bipolar/hypomania |
| Feeling loved (2 weeks) | 0.24 | 0.024 (0.010) | Quality of life |
| Recklessness (BDQ) | 0.23 | 0.046 (0.011) | Bipolar/hypomania |
| Functional impairment/Hospitalisation | 0.20 | 0.063 (0.045) | Bipolar/hypomania |
| Unstable relationships | 0.19 | 0.024 (0.010) | Emotional instability |
| Social activity | 0.16 | 0.026 (0.012) | Bipolar/hypomania |
| Duration of social phobia | 0.16 | 0.010 (0.002) | History/comorbidities |
| Motivation | 0.13 | 0.020 (0.012) | Bipolar/hypomania |
| Past elevated mood (likely) | 0.12 | 0.038 (0.017) | Bipolar/hypomania |
| Psychotic delusions | 0.11 | 0.028 (0.017) | Bipolar/hypomania |
| Sexual activity | 0.11 | 0.021 (0.011) | Bipolar/hypomania |
| Fear of death | 0.10 | 0.010 (0.003) | History/comorbidities |
| Restlessness | 0.09 | 0.019 (0.003) | History/comorbidities |
| Trust | 0.08 | 0.023 (0.010) | Personality |

**Supplementary Table 3. Top predictors of bipolar disorder across models based on the online questionnaire data.** Table shows 30 most frequently selected features and their importance (i.e. gain). BDQ=bipolar disorder questionnaire. HMQ=hypomania questionnaire. MDD=major depressive disorder. PQ=personality disorder questionnaire. SD=standard deviation.

|  | **Baseline MDD** | **Baseline low mood** | | **Baseline BD** |
| --- | --- | --- | --- | --- |
|  | **126 BD vs. 187 MDD** | **98 BD vs. 112 MDD** | **98 BD vs. 120 low mood** | **45 BD** |
| **AUROC** | 0.50 (0.34-0.62) | 0.51 (0.44-0.57) | 0.49 (0.43-0.56) | NA |
| **AUPRC** | 0.42 (0.31-0.56) | 0.47 (0.41-0.54) | 0.45 (0.40-0.51) | NA |
| **Accuracy** | 0.54 (0.41-0.63) | 0.51 (0.45-0.57) | 0.51 (0.45-0.56) | NA |
| **Sensitivity** | 0.32 (0.04-0.60) | 0.33 (0.07-0.58) | 0.33 (0.07-0.58) | 0.29 (0.04-0.56) |
| **Specificity** | 0.68 (0.45-0.92) | 0.67 (0.47-0.95) | 0.66 (0.44-0.96) | NA |
| **PPV** | 0.40 (0.23-0.56) | 0.47 (0.38-0.58) | 0.44 (0.32-0.54) | NA |
| **NPV** | 0.60 (0.50-0.69) | 0.54 (0.49-0.58) | 0.55 (0.50-0.59) | NA |

**Supplementary Table 4. Out-of-fold performance of models based on the biomarker data.** Values are shown as mean (95% confidence intervals). AUPRC=area under the precision-recall curve. AUROC=area under the receiver operating characteristic curve. BD=bipolar disorder. MDD=major depressive disorder. NA=not applicable. NPV=negative predictive value. PPV=positive predictive value.

| **Feature** | **Frequency** | **Importance, mean (SD)** | **Category** |
| --- | --- | --- | --- |
| A2MG_AIGYLNTGYQR | 0.73 | 0.096 (0.195) | Biomarker |
| IGHA1_TPLTATLSK | 0.70 | 0.062 (0.040) | Biomarker |
| PNPH_VFGFSLITNK | 0.58 | 0.077 (0.149) | Biomarker |
| C4BPA_EDVYVVGTVLR | 0.56 | 0.053 (0.065) | Biomarker |
| CO8A_AMAVEDIISR | 0.56 | 0.092 (0.190) | Biomarker |
| HBG1_MVTAVASALSSR | 0.53 | 0.031 (0.027) | Biomarker |
| CO4A_DFALLSLQVPLK | 0.52 | 0.044 (0.039) | Biomarker |
| CLUS_ELDESLQVAER | 0.51 | 0.068 (0.148) | Biomarker |
| A1AT_SVLGQLGITK | 0.47 | 0.042 (0.040) | Biomarker |
| PEDF_ELLDTVTAPQK | 0.47 | 0.029 (0.021) | Biomarker |
| AMBP_TVAACNLPIVR | 0.46 | 0.033 (0.028) | Biomarker |
| CO8B_SGFSFGFK | 0.46 | 0.072 (0.157) | Biomarker |
| KPYR_GDLGIEIPAEK | 0.43 | 0.041 (0.043) | Biomarker |
| ITIH4_GPDVLTATVSGK | 0.42 | 0.036 (0.038) | Biomarker |
| THIO_VGEFSGANK | 0.42 | 0.029 (0.029) | Biomarker |
| IF4B_SILPTAPR | 0.39 | 0.034 (0.056) | Biomarker |
| AACT_EIGELYLPK | 0.38 | 0.063 (0.162) | Biomarker |
| H4_VFLENVIR | 0.38 | 0.021 (0.016) | Biomarker |
| IGHG3_DTLMISR | 0.38 | 0.028 (0.021) | Biomarker |
| A1AT_LSITGTYDLK | 0.37 | 0.023 (0.025) | Biomarker |
| APOA1_ATEHLSTLSEK | 0.37 | 0.056 (0.161) | Biomarker |
| APOC1_EFGNTLEDK | 0.36 | 0.038 (0.048) | Biomarker |
| CO3_AGDFLEANYMNLQR | 0.36 | 0.038 (0.031) | Biomarker |
| CO4A_VLSLAQEQVGGSPEK | 0.36 | 0.034 (0.040) | Biomarker |
| HEMO_NFPSPVDAAFR | 0.36 | 0.046 (0.052) | Biomarker |
| APOC3_GWVTDGFSSLK | 0.35 | 0.031 (0.029) | Biomarker |
| IGHG1_FNWYVDGVEVHNAK | 0.35 | 0.019 (0.013) | Biomarker |
| AMBP_ETLLQDFR | 0.34 | 0.029 (0.022) | Biomarker |
| LEG3_IALDFQR | 0.34 | 0.018 (0.021) | Biomarker |
| APOH_EHSSLAFWK | 0.32 | 0.020 (0.011) | Biomarker |

**Supplementary Table 5. Top predictors of bipolar disorder across models based on the biomarker data.** Table shows 30 most frequently selected features and their importance (i.e. gain). Features are labelled as ‘UniProtKB protein ID_target peptide sequence’. SD=standard deviation. UniProtKB=UniProt Knowledgebase.

**Supplementary Figure 1. SHAP analysis of top predictors.** Figure shows the impact of the 30 most frequently selected variables (vertical axis, ordered by occurrence) on model output (horizontal axis). SHAP values above zero favour the diagnosis of bipolar disorder, and SHAP values below zero favour the diagnosis of major depressive disorder. Data points represent SHAP values averaged across the training models in which the features occurred. Data points are coloured by raw feature value. Grey points represent instances where a question was not applicable, due to the adaptive character of the online questionnaire. Values on the left show mean absolute SHAP value for each feature. Biomarkers are labelled as ‘UniProtKB protein ID (target peptide sequence)’. APOA1=apolipoprotein A1. BDQ=bipolar disorder questionnaire. HMQ=hypomania questionnaire. IGHG1=immunoglobulin heavy constant gamma 1. KNG1=kininogen 1. PQ=personality disorder questionnaire. SD=standard deviation. SHAP=SHapley Additive exPlanations. TSP1=thrombospondin 1. UniProtKB=UniProt Knowledgebase.

**Supplementary Figure 2. Example learning curves.** Plots represent model training within a single iteration of nested cross-validation using cross-validated AUROC as an evaluation metric. Model training was performed as described in Materials and Methods. Additionally, for the purpose of this figure, random sampling of the training data was applied at every boosting iteration (see legend for the random sample sizes) to assess sample-size related bias. Training set size of 250 represents the full training set. AUROC=area under the receiver operating characteristic curve.

**Supplementary Figure 3. Model performance for differently sized training sets.** Shown are mean training and test AUROCs (dots) with 95% confidence intervals (shaded areas). For each training set size (N=10, 20, 50, 100, 150, 200 and 250), nested cross-validation was performed as described in Materials and Methods, wherein additionally the indicated subsample of the full training data was randomly selected at each training step. Training set size of 250 represents the full training set. Test set size was fixed at N=63, i.e. 20% of the full dataset. AUROC=area under the receiver operating characteristic curve. CV=cross-validation.
